# Assessment of critical mineral extraction from brines at Mount Meager, Southwestern BC, Canada

**DOI:** 10.1038/s41598-025-01044-9

**Published:** 2025-10-06

**Authors:** Fateme Hormozzade Ghalati, Dariush Motazedian, James A. Craven, Stephen E. Grasby, Victoria Tschirhart

**Affiliations:** 1 Department of Earth Science, Carleton University, Ottawa, ON K1S 5B6 Canada; 2 Geological Survey of Canada, Ottawa, ON K1A 0E8 Canada; 3 Geological Survey of Canada, Calgary, AB T2L 2A7 Canada

**Keywords:** Critical mineral extraction, Lithium, Geothermal reservoir, Brine concentration, Mount Meager, Geochemistry, Geothermal energy

## Abstract

Critical minerals, essential for the development and sustainability of clean energy technologies, are typically sourced through conventional CO_2_ intensive mining methods. This paper evaluates the potential of geothermal brines as a sustainable alternative for mineral extraction after geothermal energy production. A detailed case study of a Canadian geothermal field provides insight into the potential economic advantages of mineral extraction from brines. Water chemistry data from the Mount Meager geothermal field, which has one of the highest geothermal potentials in Canada, demonstrates that the fluids are rich in dissolved minerals and metals. Using reservoir physical information, Monte Carlo simulations, and appropriate probability distributions, our study addresses uncertainties in volumetric resource calculations. Taking into consideration flow pathways through the rock matrix, and available technologies with rates of mineral recovery up to 90%, results show promising reserves for minerals such as lithium, magnesium, and silica. The findings highlight the dual benefits of geothermal energy in Canada providing both a green energy source and facilitating critical mineral production. This dual utility can generate additional revenue fostering the development of geothermal fields, even those that are not viable for energy generation on their own, supporting Canada’s transition to a low-carbon economy.

## Introduction

Critical minerals are non-fuel minerals, elements, substances, or materials with (1) a high risk for supply chain disruption, and (2) have a crucial function in one or more energy technologies, such as the production, transmission, storage, and conservation of energy^1^. Because most critical minerals are crucial for clean energy technologies and industries, like electric vehicles, they are key to achieving a low-carbon economy. In Canada, there are currently 34 elements and minerals listed as critical, including aluminum (Al), lithium (Li), cobalt (Co), silica (Si), and rare earth elements (REEs) like dysprosium (Dy) and neodymium (Nd). Canada seeks to integrate environmental, social and governance considerations, including Indigenous perspectives, into its mineral resource strategy by recognizing its rich mineral endowment across diverse regions^2,3^. These mineral resources are normally extracted from geological formations through conventional mining methods^4–7^.

Conventional mining techniques are associated with significant environmental challenges, and contribute to greenhouse gas emissions^7^. The mining industry contributes to approximately 11% of total carbon dioxide (CO_2_) emission in Canada and 8% of the global CO_2_ footprint^8,9^. Alternatively, mineral extraction from fluids presents a more sustainable choice due to low energy consumption and carbon emissions^10^. One source of fluid is from geothermal resources, which can contain critical minerals and even precious metals like silver, gold, palladium, and platinum^11^.

Geothermal resources can provide electricity and thermal energy for various applications by drilling boreholes to access hot water. After energy production, the remaining fluids are typically re-injected into the reservoir to maintain reservoir pore pressure by flowing through permeable zones and fracture networks^12^. However, these fluids also offer opportunities for mineral extraction before re-injection, as they are rich in dissolved metals and other elements such as lithium, silica, and magnesium, due to contact with subsurface rocks. This process, akin to natural solution mining, can be followed by hydrometallurgical techniques to isolate and purify minerals^13–15^. Solution mining can substantially reduce CO_2_ emissions over traditional mining practices. Studying minerals in brines has gained recent attention by different countries as emerging environmentally friendly technologies are advancing (Table 1)^7,16–26^. For instance, it is projected that the Salton Sea geothermal field will produce more than 600,000 tons of lithium carbonate annually, representing to a potential value of USD 7.2 billion^27^. Additionally, as part of the U.S. Geothermal Technologies Program, efforts are underway to develop cost-effective and environmentally sustainable techniques for extracting silica from geothermal fluids. This project aims to both produce commercially viable silica and prevent silica scaling at three geothermal locations: Dixie Valley and Steamboat Springs in Nevada, and Coso in California. A range of methods, including precipitation and adsorption techniques, are being applied depending on the target elements^13^.

**Table 1:** Summary of the critical elements (in parts per million or ppm) in major geothermal fields in the world^28–37^.

	Salton Sea (USA)	Coso (USA)	Dixie (USA)	Wairakei (NZ)	Kawerau (NZ)	Simav (Turkey)	Soultz- Forêts (France)
Temperature (°C)	296	274	246	260	310	190	150
Boron (ppm)	257	119	9.9	27	57	3.56	*
Lithium (ppm)	194–230	45	2–4	12.7	6.3	1.76	150
Silica (ppm)	461	711	599	618	954	281	189
Zinc (ppm)	438	0.03	*	0.002	0.35	5.5	3
K (ppm)	12,000	928	64	194	119	62.7	3200
Mg (ppm)	19	< 0.35	0.007	0.04	0.01	0.42	124
Cesium (ppm)	20	*	*	2.5	0.6	0.02	14
Rubidium (ppm)	170	*	*	2.9	0.7	0.01	22

* Data not available.

Geothermal energy prospects, with a potential for solution mining, occur over broad regions of Canada. Geothermal resources within Canadian sedimentary basins have moderate to low temperatures; however, temperature anomalies (up to 150 °C) at depths shallower than 3 km demonstrate electrical generation potential in northeastern British Columbia (BC), Alberta, and southern Northwest Territories^38^. Heat flow in the Canadian Cordillera is similar to that of the western United States of America’s (USA) Basin and Range region, where most global geothermal power is produced^39,40^. Within the Canadian Cordillera, heat flow > 200 mW/m^2^, the highest recorded in Canada, occurs in the Mount Meager geothermal field of the Garibaldi Volcanic Belt (GVB).

Mount Meager, the focus of this paper, is a Quaternary volcanic complex located between the Lillooet River and Meager Creek. While previous studies have explored the geothermal potential at Mount Meager, most have focused on the geological features and the possible mechanisms and size of the reservoir^41–43^. However, there has been limited evaluation of the presence and potential for extraction of critical minerals. Due to the elevated concentrations of critical elements in the geothermal fluids, it is important to assess Mount Meager’s potential, not only as one of Canada’s most promising geothermal sites, but also as a source of critical mineral resources. In this study, Monte Carlo simulation, which is a statistical approach, is used to model geochemical data of Mount Meager. This technique utilizes a range of probability distributions that approximately reflect the actual distributions of input variables, thereby capturing the inherent variability and uncertainty in the system. It accounts for the stochastic nature of each input parameter through repeated iterations of random sampling^44^. Unlike deterministic models, which rely on fixed input values, Monte Carlo simulation generates various realizations that reflect the heterogeneity of the data^45,46^. Using this approach, we investigate the potential of geothermal energy to support critical mineral extraction and evaluate the resource potential of the Mount Meager geothermal system.

## Study area

**Fig. 1:**
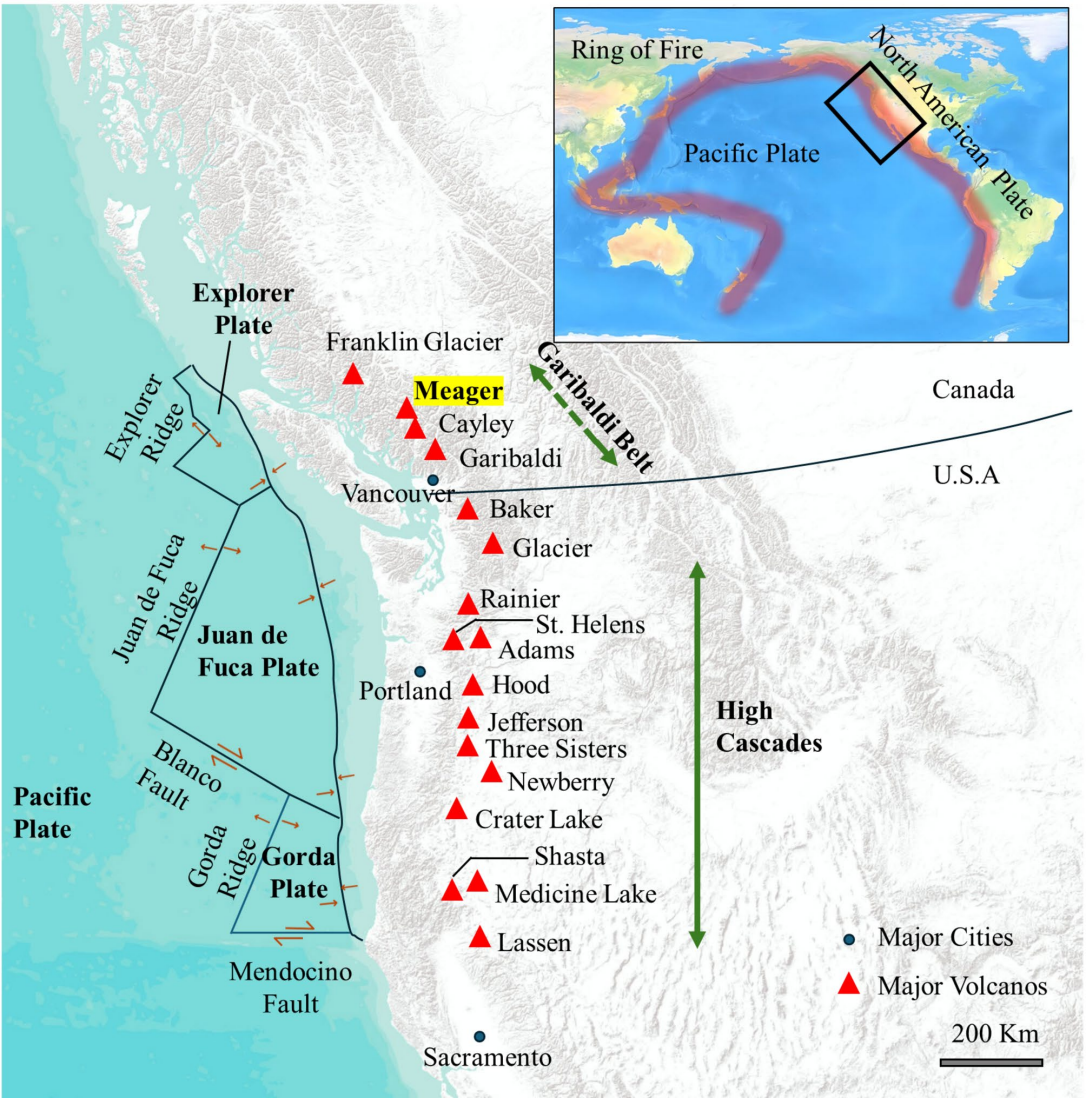
The inset illustrates the broader Ring of Fire, with the black square highlighting the region depicted in the main map. The main map shows the North American side of the Ring of Fire (Cascade volcanic arc) and its tectonic setting. Major volcanoes are shown in red rectangles. The extent of the High Cascades and the GVB segments of the arc are indicated with green lines. Mount Meager is highlighted in yellow within the GVB (modified from^43^). This figure is created using ArcGIS 10.8 (GIS Software for Mapping and Spatial Analytics | Esri).

Geothermal systems are normally found at plate boundaries with large faults and volcanoes, such as the Ring of Fire where the Pacific plate subducts beneath continental crust (top-right insert in Fig. 1). The Cascade Volcanic Arc is a prominent geological feature in the Pacific Northwest, extending from northern California through Oregon and Washington in the USA up to western British Columbia in Canada. As shown in Fig. 1, this volcanic arc is primarily formed due to the subduction of the Juan de Fuca Plate beneath North American in a north-easterly direction, resulting in enhanced thermal gradients in the region^47–49^. Geochemical evidence along the Cascade Volcanic Arc suggests that magmas originate from varying depths and the melt formation process involves complex mantle-crust interactions contributing to the formation of numerous volcanoes, thermal springs, and the high heat flow associated with volcanic activity^50^.

The central and southern segment of the Cascade Arc, including volcanoes such as Mount Adams, Mount St. Helens (with an eruption in 1980), are known for their geothermal potential^51^. The northern segment of the Cascade Arc is the GVB in southwestern British Columbia with volcanic deposits range from Pleistocene to Holocene^52^. The GVB includes various flows, domes, landforms, and stratovolcanoes such as Mount Cayley, Mount Garibaldi, and Mount Meager.

**Fig. 2:**
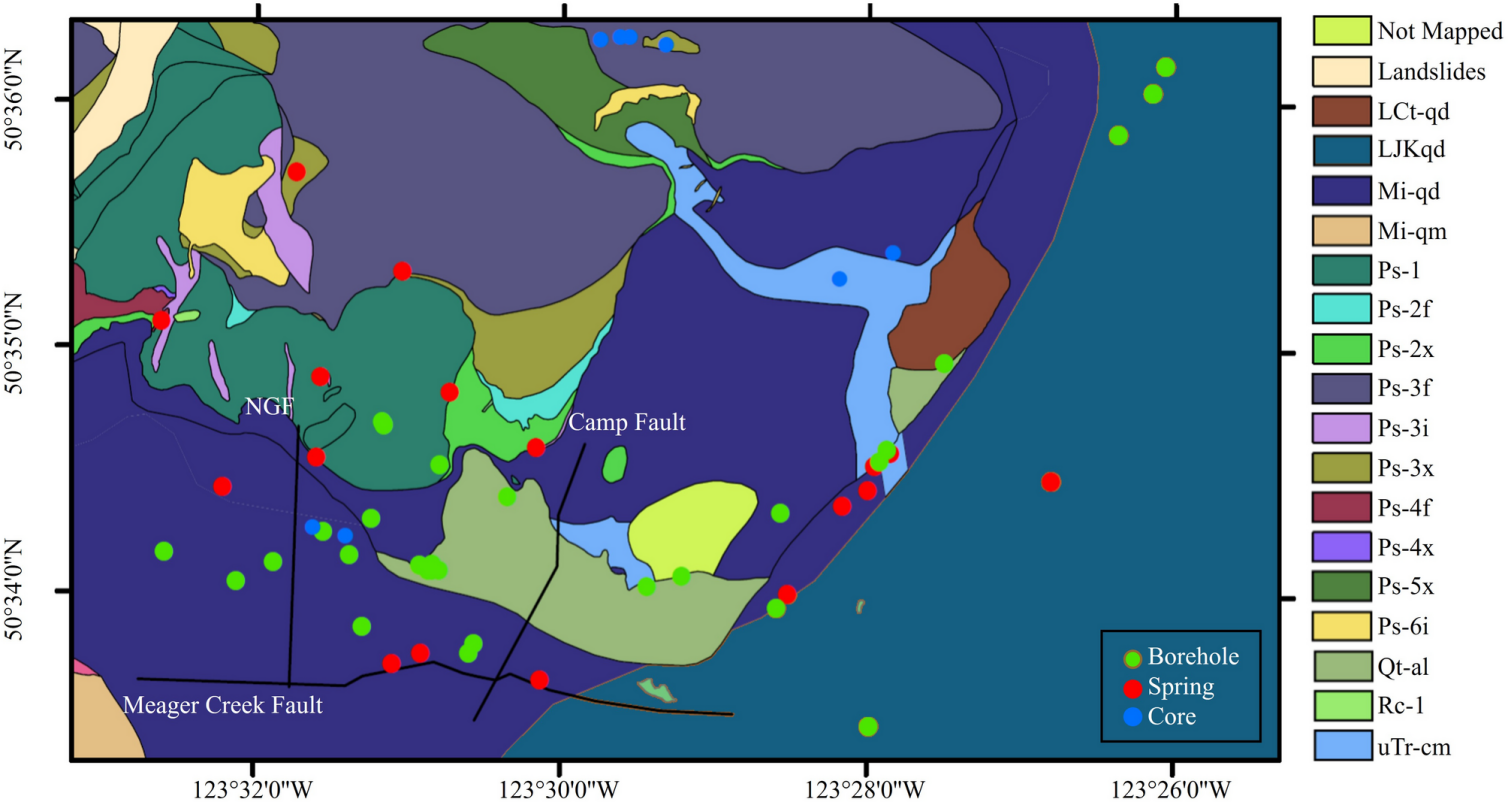
An overview geological map of the Mount Meager volcanic complex showing the distribution of rock units (created from data in^62,63,65^), as well as the locations of core samples, boreholes, thermal springs, and local faults. Rock unit abbreviations: LCT-qd– Late Cretaceous quartz diorite intrusive rocks; Mi-qd– Biotite hornblende quartz diorite; Mi-qm– Biotite quartz monzonite; Ps-1– White, altered rhyodacite tuff, breccia and flows; Ps-2f– Dark grey, aphanitic andesite flows; Ps-2x– Volcanic breccia with dominant plutonic and some aphanitic volcanic clasts; Ps-3f– Porphyritic (plagioclase ± pyroxene) andesite flows; Ps-3i– Porphyritic (plagioclase ± pyroxene) andesite; Ps-3x– Porphyritic (plagioclase ± pyroxene) andesite breccia and ash; Ps-4f– Porphyritic (hornblende, plagioclase) andesite flows; Ps-4x– Porphyritic (hornblende, plagioclase) andesite breccia; Ps-5x– Porphyritic (hornblende, plagloclase ± biotite) andesite breccia and tuff; Ps-6i– Light grey, porphyritic (plagioclase, hornblende) andesite; Qt-al– Alluvium; few if any outcrops; Rc-1– Blocks and ash of porphyritic (plagioclase, quartz, hornblende, pyroxene, biotite) rhyodacite pumice; uTrcm– Streaky amphibolite. This figure is created using ArcGIS 10.8.1 (GIS Software for Mapping and Spatial Analytics | Esri).

Mount Meager, the focus area of this paper, is located about 150 km north of Vancouver. Recent volcanic activity, from 2.2 Ma (based on K–Ar age dates) to the most recent explosive eruption approximately 2,350 years ago, produced a sequence of rhyodacite volcaniclastic deposits and lava domes^53,54^. Various studies using geophysical, geological, and geochemical methods, as well as a small-scale test power production, confirmed a geothermal reservoir in Mount Meager^55–57^. These studies have examined the geological setting and structural features of the Mount Meager volcanic complex, which includes three major faults of Meager Creek Fault, No-Good Fault (NGF), and Camp Fault, as shown in Fig. 2^58–61^. The complex has been subdivided into various assemblages, each emplaced during Pliocene to Pleistocene rhyodacite, early to late Pleistocene dacite to andesite-basalt, and Late Pleistocene to Holocene and recent rhyodacite to dacite composition^62–64^. Our survey includes various rock units as shown in Fig. 2.

Research on geothermal potential and hydrothermal alteration has provided insights into fluid circulation and reservoir characteristics^61,66^. Most of the rocks associated with the water sampling locations in this study are altered (zeolite, sericite, chlorite, and epidote), with granodiorite, andesite, and quartz diorite being abundant. While core samples showed a porosity range of 2.6–23.2% and permeability range of 0.001–5,186.57 millidarcies (mD), modeling of potential reservoir show a porosity of up to 8.5% and permeability of approximately 0.25 mD. Additionally, the rocks are highly fractured due to past explosive eruptions^43^, in addition to the presence of local faults. These provide flow pathways for thermal fluid, with measured temperature exceeding 250 °C within the first 2 km below the surface^43^. These observations suggest that Mount Meager hosts an active hydrothermal system with potential fluid-rock interactions^40,59^.

## Data and methodology

**Fig. 3:**
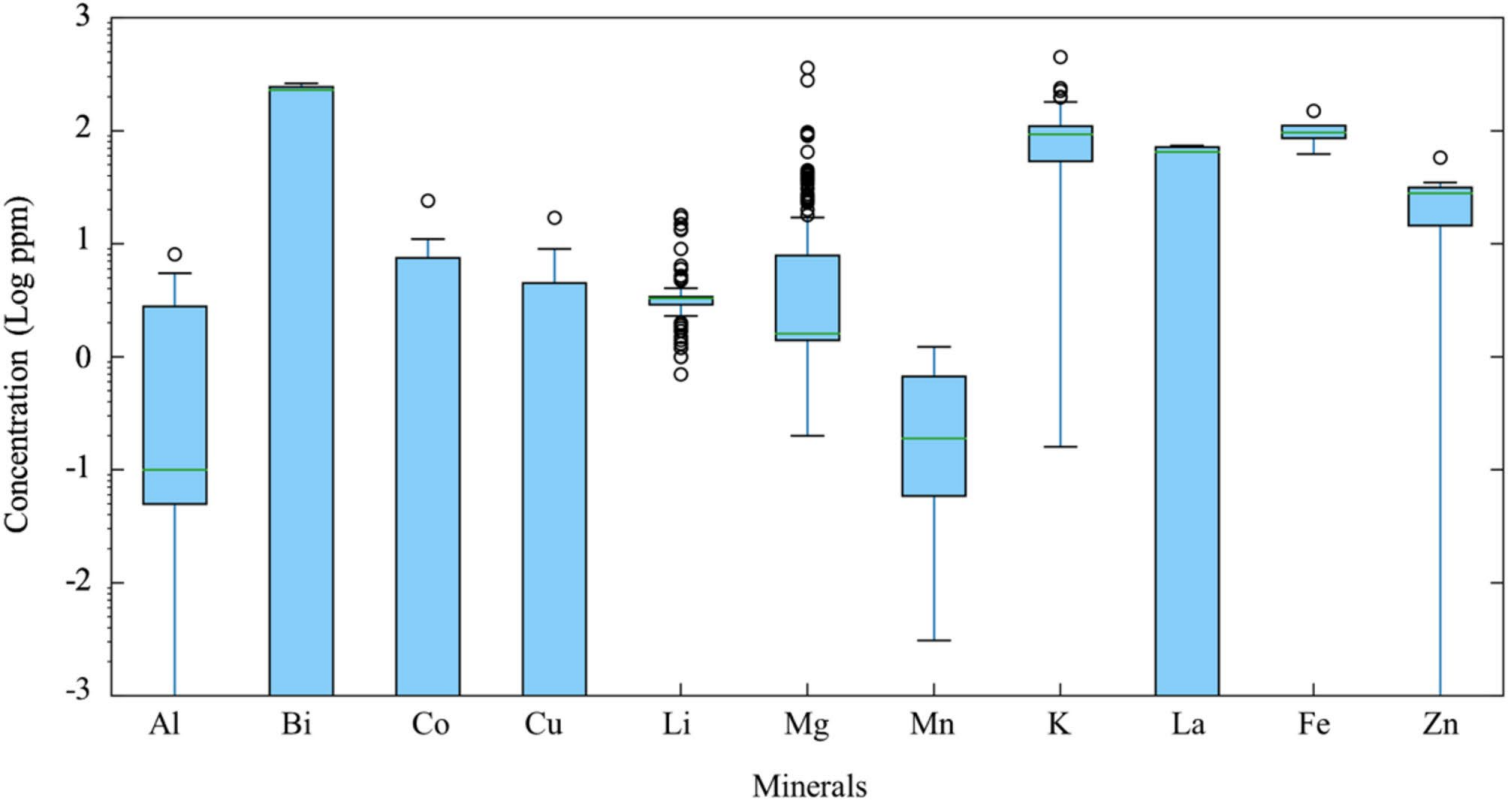
Box plot of Mount Meager geothermal fluid composition for major critical minerals.

We compiled chemical compositions collected from exploration and research boreholes, surface water, and thermal springs at the Mount Meager geothermal field from old reports, recent water analyses, and available journal articles^41,55,60,61,67^. Figure 3 shows the range of concentrations for each of the measured critical elements in the Mount Meager geothermal fluids, ranging from 1 ppt up to hundreds of ppm, depending on the element.

The methodology employed in this study is based on Monte Carlo simulations to estimate the mineral contents with volumetric calculations. Monte Carlo simulation has been widely applied in the assessment of geothermal resources potential, estimation of energy capacity, and economical evaluation of geothermal systems^44,68^. The Monte Carlo method, renowned for its robustness in handling uncertainties, was chosen due to the natural variability and uncertainty in reservoir parameters. Parameters considered in the volumetric calculation are shown in Eq. (1) and include the reservoir surface area (A in km^2^), reservoir thickness (h in m), porosity (φ as a fraction), element concentration (C in ppm), recovery factor (RF as a fraction), and efficiency (E as a fraction)^35,69^.

1 $$Mineral{\text{ }}Content = A \times h \times \varphi \times C \times RF \times E$$

By sampling from the distributions of these uncertain parameters, we generated 10,000 random realizations for the mineral content and created a discrete probability distribution of possible results. This is achieved by modeling each uncertain parameter with a triangular distribution, which is effective when the range of data and the most probable value is known. The probability density function for a triangular distribution, where a is the minimum value, b is the maximum value, and c is the most likely value, is defined as:

2 $$\:f\left(x\right)=\left\{\begin{array}{c}0,\:\:\:\:\:\:\:\:\:\:\:\:\:\:\:\:\:\:\:\:\:\:\:\:\:\:\:\:\:\:\:\:\:\:\:\:\:\:\:\:\:\:\:for\:x<a\\\:\begin{array}{c}\frac{2\left(x-a\right)}{\left(b-a\right)\left(c-a\right)}\:\:\:\:\:\:\:\:\:\:\:\:\:\:\:\:\:\:\:\:\:\:\:\:for\:a\le\:x<c\\\:\frac{2\left(b-x\right)}{\left(b-a\right)\left(b-c\right)}\:\:\:\:\:\:\:\:\:\:\:\:\:\:\:\:\:\:\:\:\:\:\:for\:c\le\:x<b\end{array}\\\:0,\:\:\:\:\:\:\:\:\:\:\:\:\:\:\:\:\:\:\:\:\:\:\:\:\:\:\:\:\:\:\:\:\:\:\:\:\:\:\:\:for\:x\ge\:b\end{array}\right.$$

This Monte Carlo simulation helps in identifying the influence of each parameter on the outcome and in understanding how variations in the input parameters can affect the mineral estimates^70^. Each of the parameters in Eq. (1) were modeled using a triangular distribution to capture their range of possible values and their potential results, which is specifically effective in modeling uncertainties in sparse data^71^.

Since Mount Meager is still under study, the RF and E values were chosen based on a review of existing geothermal mineral extraction studies. RF is the amount of energy that is extractable from the reservoir, which is related to the reservoir’s fluid and rock physical properties^72^. Various studies have defined RF for geothermal reservoirs production and showed a range from 10 to 20%^72–75^. The efficiency factor depends on the method and technology that is used for mineral extraction^76^. Disu et al. summarized lithium recovery efficiency of calcium-, sodium-, and aluminium-based precipitation systems applied to various lithium resources^76^. They reported recovery rates of 99.3–99.57% and purity of 99% using a combination of CaO and Na_2_CO_3_. Mineral extraction efficiency also varies with the composition of saline^76^. Therefore, extraction of different minerals from thermal water has different E values with ranges from 50 to 90%^7,27,77^.

Porosity (φ) is the amount of pore space in a medium. A, h, and φ in Eq. 1 are related to the physical properties of the reservoir. The ranges for these parameters are derived based on the audio-magnetotelluric (AMT) method, which is a passive electromagnetic (EM) technique that measures the Earth’s natural EM field at high frequencies (~ 0.001–10,000 Hz)^78^. This technique provides detailed resistivity models that help in identifying areas with different geological systems, temperature anomalies, saline water, and partial melt within hydrothermal systems. A comprehensive AMT dataset (from 84 stations) was collected in 2019, focusing on the surface-to-reservoir depths at Mount Meager. These data were inverted to produce an electrical resistivity model down to a depth of 3 km^79^. The extent and thickness of the near surface reservoir was defined from the propylitic alteration zone beneath the potential caprock in the system. Additionally, the porosity of the potential reservoir was defined using the AMT resistivity model and Archie’s law^42,43^.

## Evaluation of critical minerals in Mount Meager

**Fig. 4:**
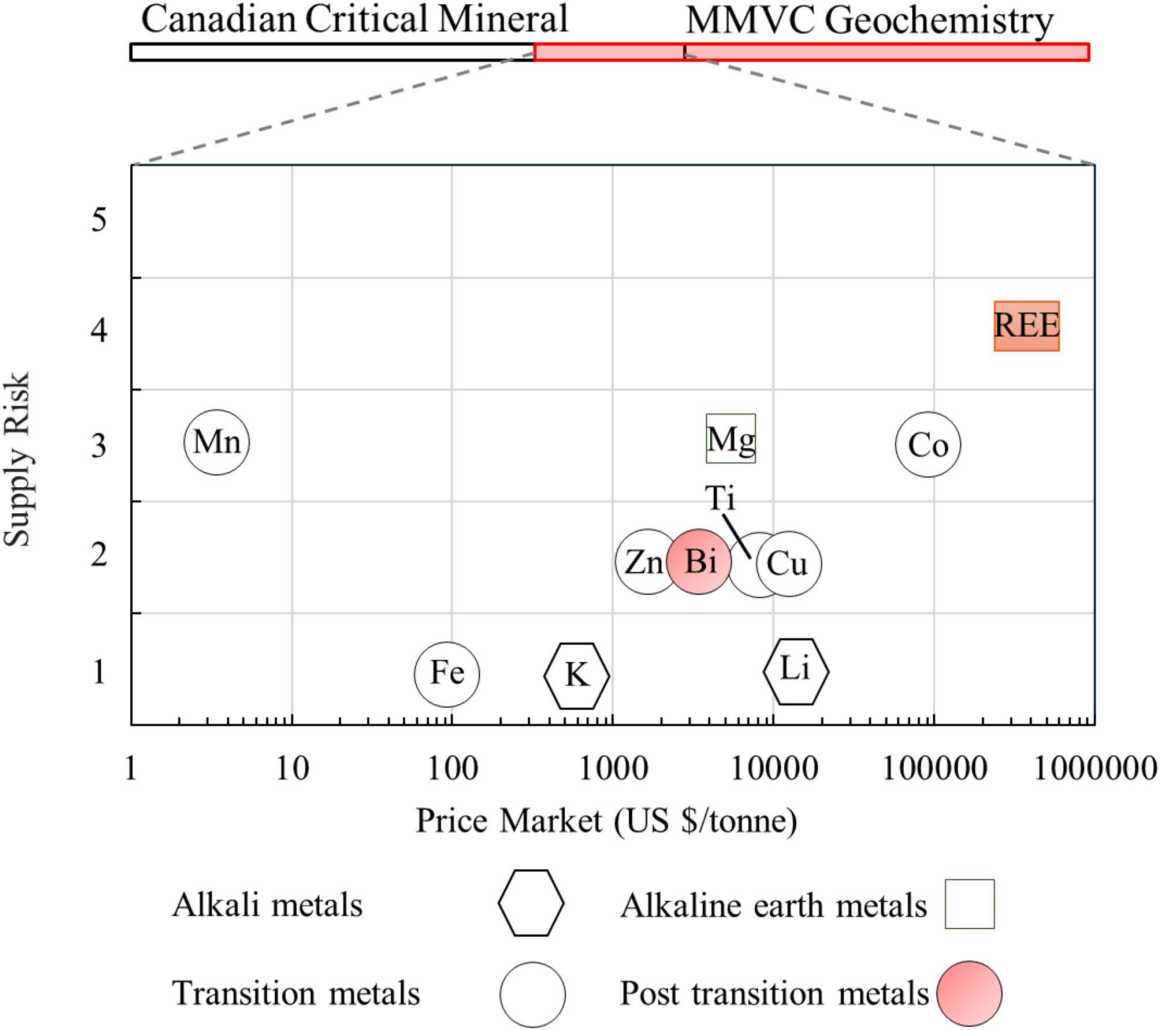
Overlap of the elements in the Mount Meager geothermal fluid and the critical minerals listed by the Canadian government. The scatter plot shows the average market price versus supply risk for these minerals^81–86^.

The interaction of volcanic activity with glaciation in the Mount Meager geothermal field has led to unique geothermal features and geochemistry^49,80^. Figure 4 shows the elements from the Canadian critical mineral list that are available and measured in Mount Meager geothermal fluids. Some of these elements with high market price and supply risk will be discussed here for Mount Meager.

**Fig. 5:**
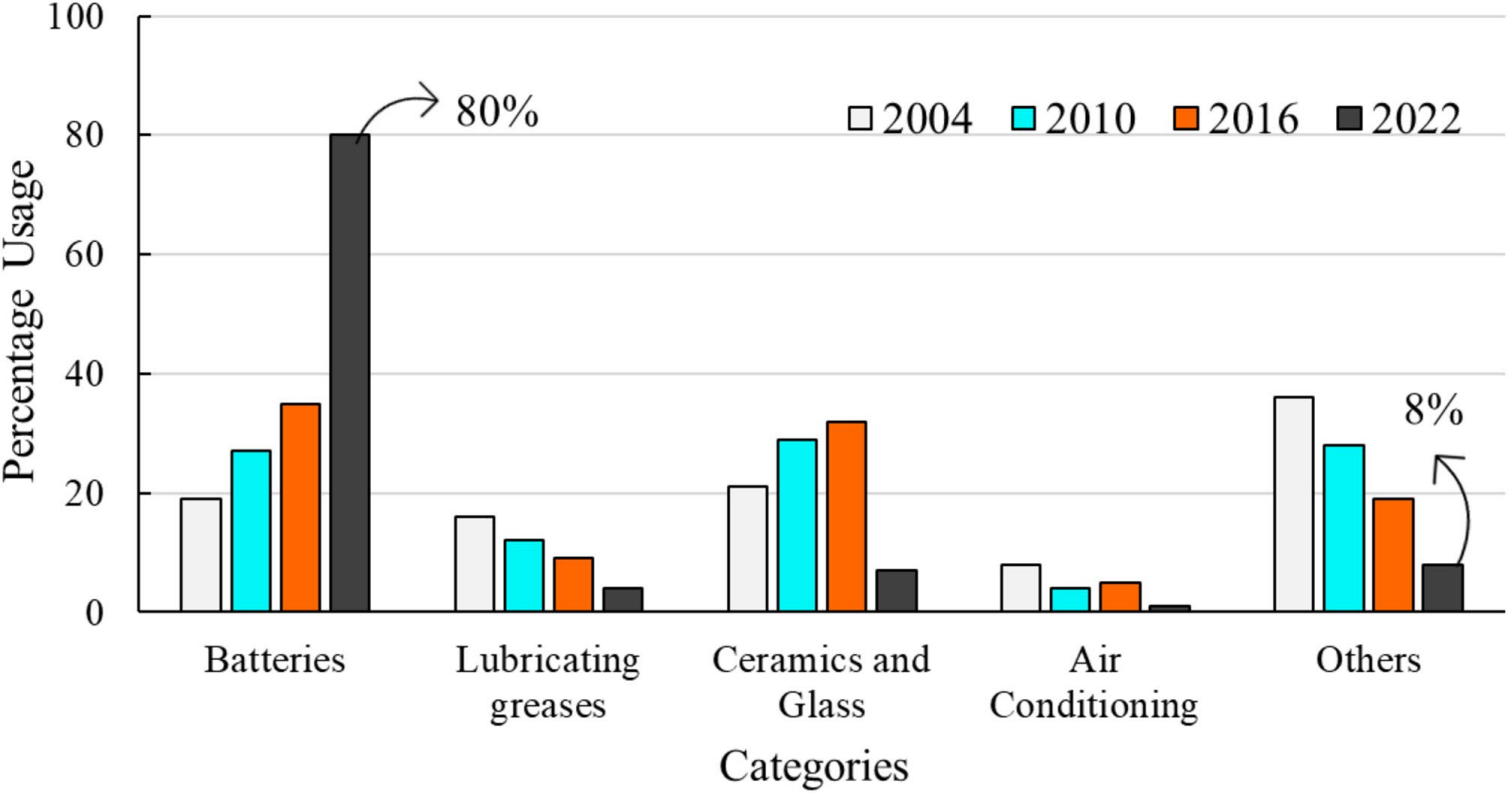
Global utilization of lithium from 2004 to 2022^87–90^.

Lithium is a critical mineral that is crucial for various clean energy technologies, and exists in fluids at Mount Meager. About 173, or 73%, of the water analyses at Mount Meager were measured for lithium concentrations with an average of 3.5 ppm and the highest amount of 18 ppm. While normal concentration of Li in seawater is about 0.17 ppm, geothermal fluids can contain lithium up to hundreds of times higher concentration^16^. Figure 5 shows major global usage of lithium in various years. Due to its increasing demand in electronic and transportation sectors, the processing of lithium has acquired extensive attention in recent years. This element is notably soluble throughout the geothermal energy production process, from reservoir extraction to reinjection^34^.

In our database, seven fluid samples from Meager Creek hot spring surface samples, have copper concentrations reaching up to 17 ppm. On the other hand, downhole samples show significantly higher concentrations of other elements, including calcium (up to 810 ppm), potassium (up to 450 ppm), and silica (up 480 ppm), which can reflect the loss or precipitation of some elements from a fluid while flowing upwards, towards the wellbore. Additionally, about 34 samples (14% of total) in our dataset have known manganese concentrations, all of which have manganese concentration values less than 1.2 ppm.

**Fig. 6:**
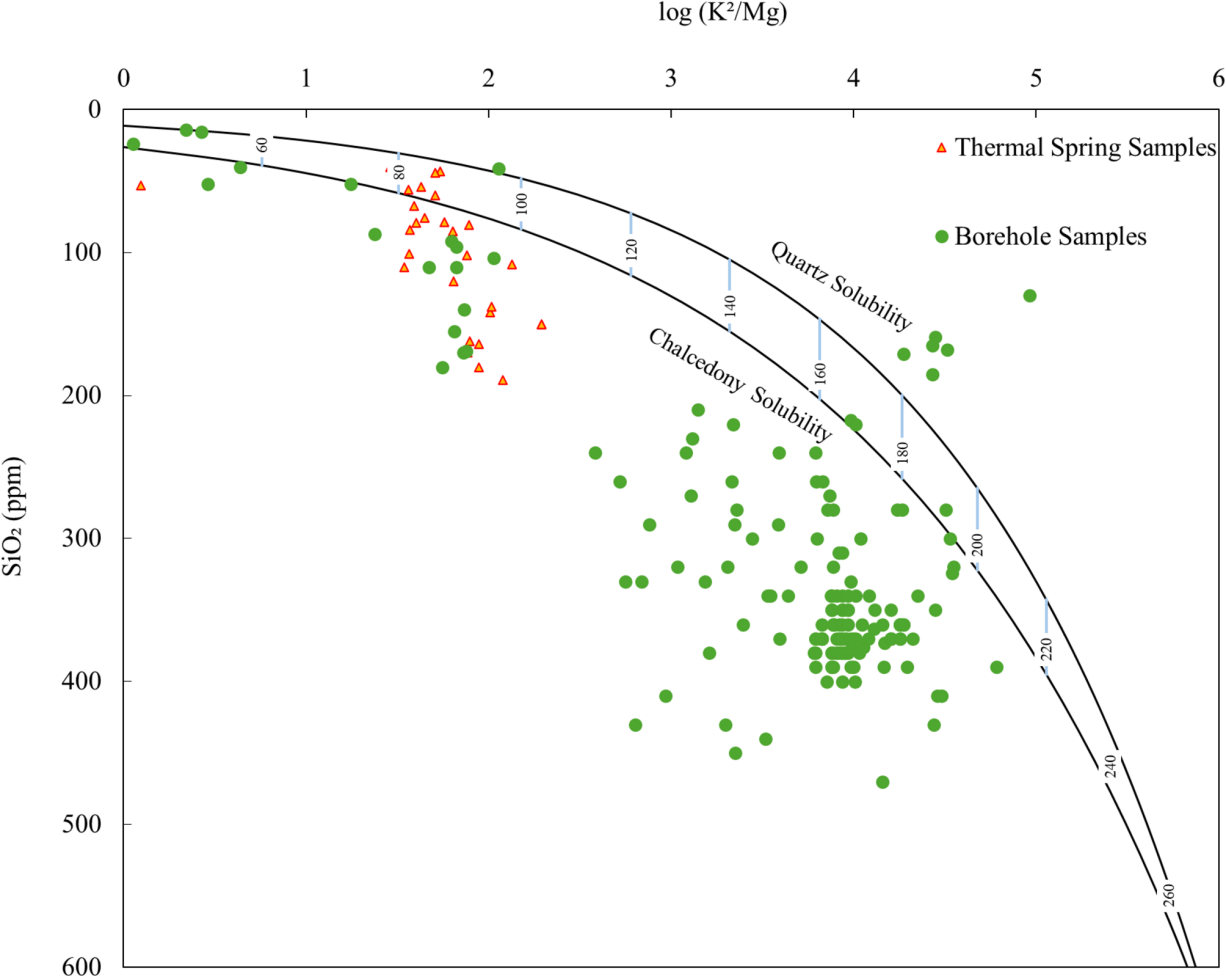
Cross plots of log (K^2^/Mg) vs. SiO_2_ concentration in water samples from boreholes and thermal springs at Mount Meager, with estimated reservoir temperatures shown as blue lines.

Silica, which is mostly in the form of silicon dioxide (SiO_2_), is abundant in the Mount Meager geothermal fluids, and over 261 samples, i.e., 96% of the samples, in the database have reported silica concentrations with an average of 270 ppm. Silica is commonly present in geothermal fluids all over the world and comes from rocks rich in quartz in Earth’s crust that dissolve in hot geothermal fluids under high pressure^91^. Because silica solubility highly depends on temperature and fluid composition, higher reservoir temperatures normally lead to higher dissolved silica concentrations and worsening scaling issues. Managing silica deposition is critical in geothermal energy production to prevent operational challenges and maintain equipment integrity. In Mount Meager, borehole samples show elevated silica concentrations, and most samples exceed quartz and chalcedony solubility limits (Fig. 6). The lines in Fig. 6 represent simultaneous attainment of equilibrium for the systems involving silica and K–Mg^92^. Samples from thermal springs and shallower zones in boreholes show a lesser amount of silica (Fig. 6). This supersaturation suggests that the fluids have been exposed to high-temperature zones (up to 200 °C), with higher water-rock interaction and potential silica precipitation. In addition to extracting silica and enhancing the economic viability of geothermal projects, silica removal can facilitate the co-recovery of valuable metals like zinc, manganese, and lithium from the brines^93^.

Rare earth elements (REEs) are normally found in geothermal fluids around the world in low concentrations, which do not exceed 1 ppb (REEs concentrations higher than 1 ppb are from acidic hot springs)^11,94^. At Mount Meager, only seven samples were tested for Lanthanum (La), and they showed an average of 70 ppm. These samples were collected at the Meager Creek hot spring. Additionally, boron, which is a critical element in most countries but is not in the Canadian critical mineral list, has a high concentration at Mount Meager that can be attributed to the argillic rocks.

## Discussion

Comparing mineral concentrations in fluids at Mount Meager (Fig. 3 and Sect. 4) with other geothermal fields worldwide that have started exploring for critical minerals in geothermal fluids, shows that Mount Meager fluids could potentially be considered economic (Table 1).

Typically, every 1 MW of geothermal energy (at 200 °C) is associated with a flow rate of about 20 kg/s of fluid^11^. Considering up to 100 MW energy capacity at Mount Meager (reported by Western GeoPower Corp in 2009), it can be estimated that circa 6.3 × 10^7^ Tonnes/year of thermal fluid would be produced. Using this production rate and the mean mineral concentrations, we can roughly estimate the potential mineral reserves at Mount Meager. Calculations show around 504 tonnes/year of magnesium, 15 tonnes/year of manganese, 13,875 tonnes/year of silica, 756 tonnes/year of iron, 151 tonnes/year of lithium, and 4,187 tonnes/year of potassium at Mount Meager, considering an extraction efficiency of about 80%.

**Fig. 7:**
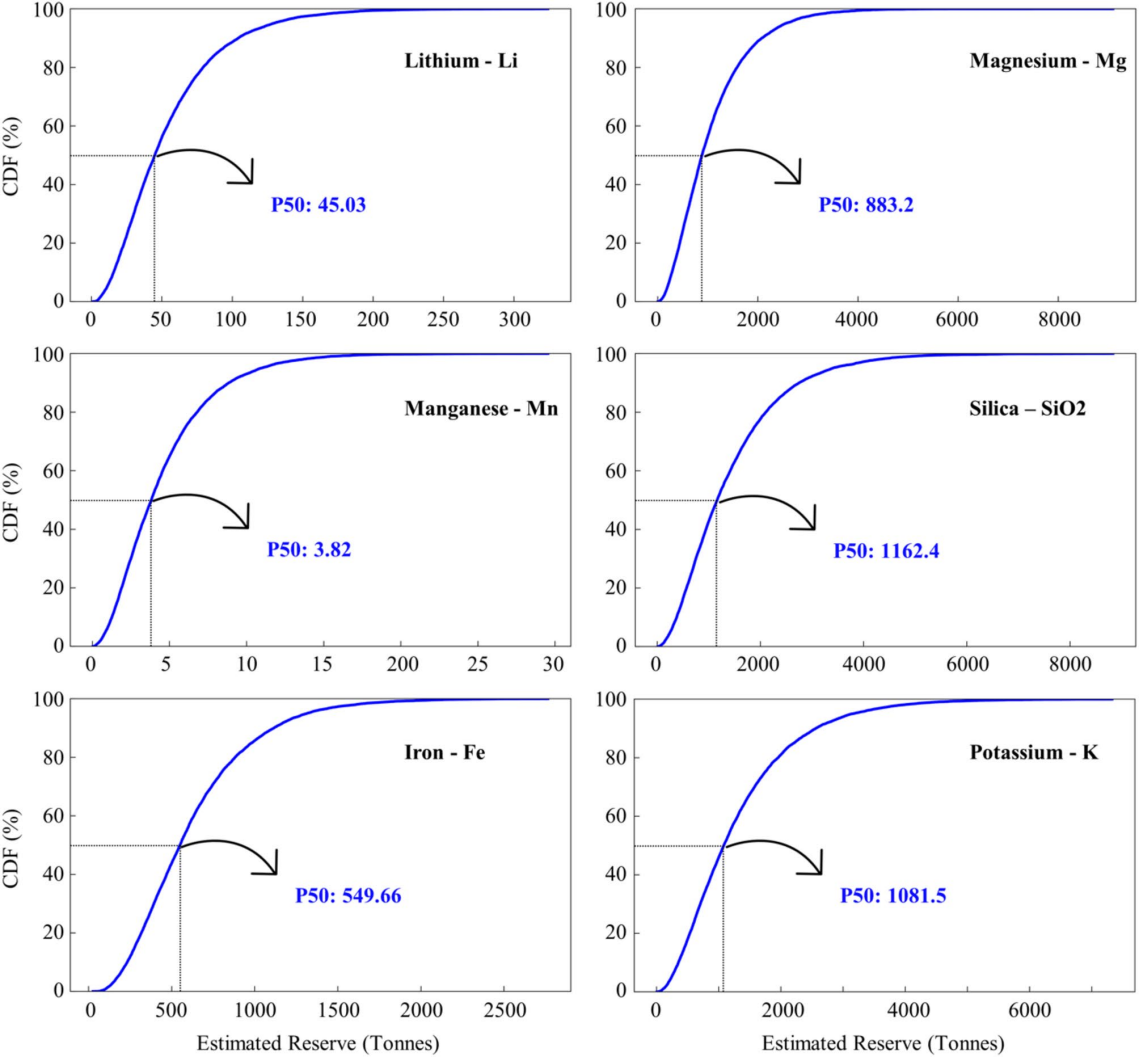
Cumulative distribution function of lithium, magnesium, manganese, silica, iron, and potassium reserves at Mount Meager.

**Fig. 8:**
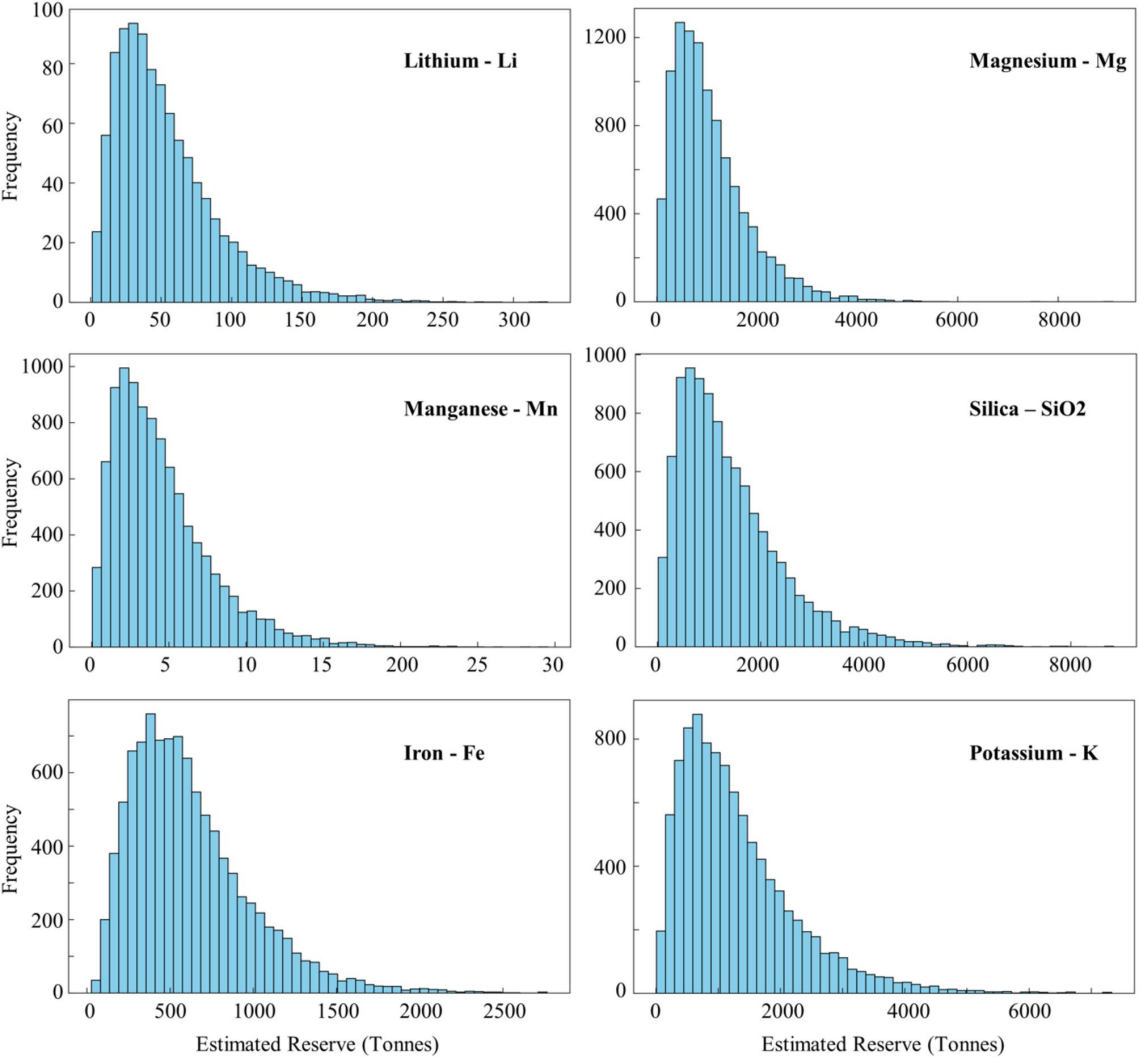
Histogram of reserve estimation for lithium, magnesium, manganese, silica, iron, and potassium at Mount Meager.

Although such deterministic calculations can be used for estimating the reserves, using a probabilistic approach can more reliably evaluate the potential of geothermal resources. After modelling potential reserves for each mineral using Eq. (1), the cumulative distribution function (CDF) of the results and histograms were plotted for each mineral to visualize the probability of achieving different mineral contents at Mount Meager (Figs. 7 and 8). The use of CDFs is particularly useful in handling uncertainties because it provides a comprehensive view of the range and likelihood of different mineral reserves, rather than relying on a single deterministic estimate. By presenting the data in this way, it is possible to understand the variability of the mineral reserves. A key percentile of P50 is identified on each plot to indicate that there is 50% possibility to achieve that amount of mineral reserve within the system. This method can provide insights into the low, median, and high estimates of the mineral contents^95^. In Mount Meager, we discovered six critical minerals with higher concentrations, and therefore, larger reserves, which are discussed below.

**Fig. 9:**
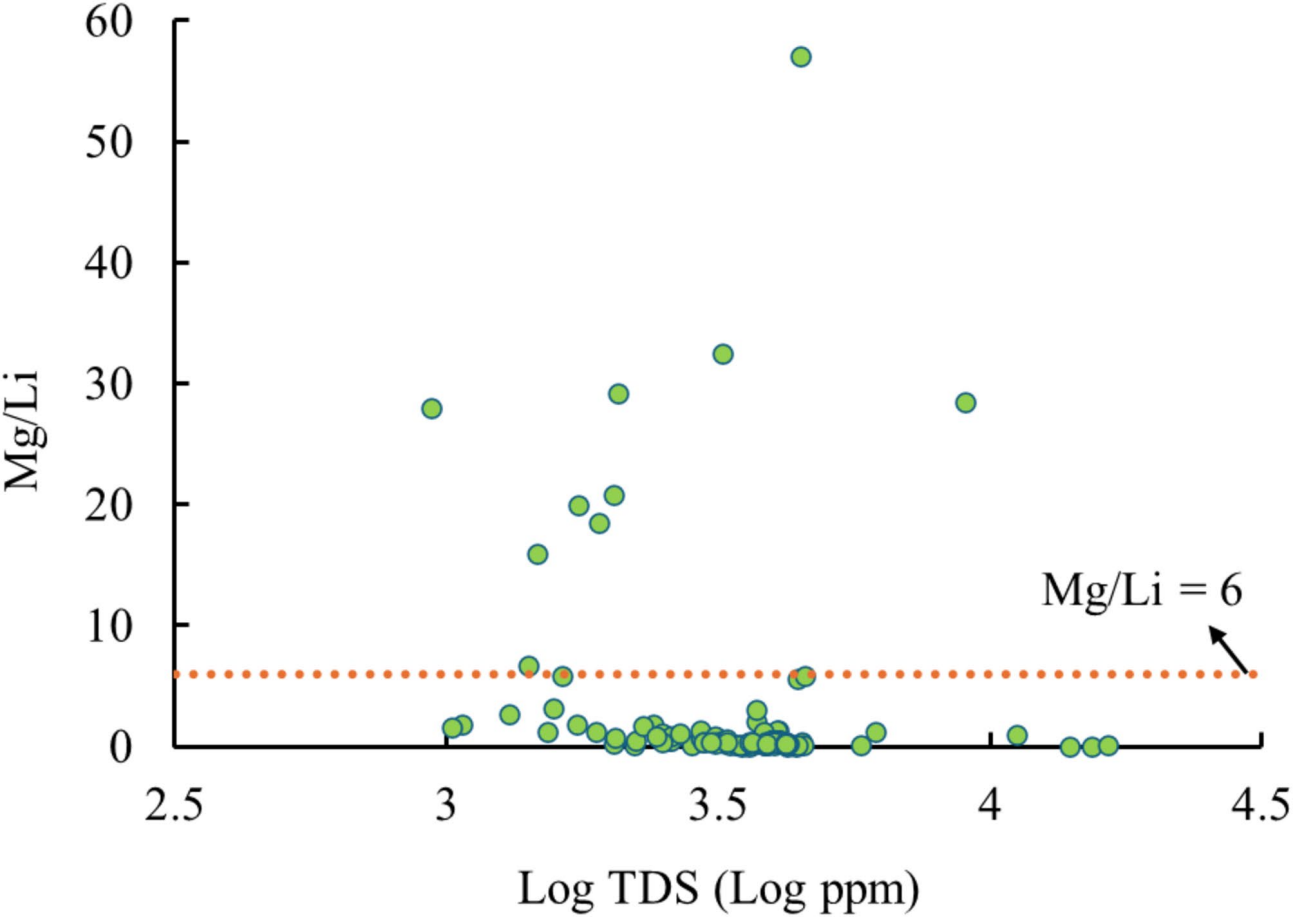
The ratio of the concentration of magnesium to lithium vs. the TDS of fluid samples from Mount Meager.

- Silica: Over 86% of the fluid samples at Mount Meager have silica concentration of more than 100 ppm. The volumetric approach suggests a 50% probability of producing 1,162 tonnes of silica per year at Mount Meager (Figs. 7 and 8). Normally, the concentration of silica increases with increasing reservoir temperature (Fig. 6). Therefore, geothermal sites with potential for power production are probable to have economic levels of silica in their fluids. Not all brines with high silica content are economically attractive though. In general, fluids containing relatively low TDS and higher level of silica produce high-value silica products with favorable economics because pre-processing of these brines to remove potential impurities can be avoided^21^.
- Magnesium: Fluid samples from Mount Meager show magnesium concentrations of up to 360 ppm. Based on these concentrations, there is a 50% likelihood of a reservoir with a potential production capacity of 883 tonnes per year.
- Lithium: In Mount Meager, fluid samples that are taken from borehole fluids have higher lithium concentrations than samples taken from thermal surface water. Although the highest measured lithium at Mount Meager was 18 ppm, Neupane and Wendt (2017) conducted a comprehensive review of over 2,250 geothermal fluid chemical analyses across the USA and found that lithium concentrations exceeding 20 ppm were rare^96^. Lithium concentrations at Mount Meager can support a reserve of 45 tonnes/year with a possibility of 50%. The efficiency of lithium extraction from brines depends on the ratio of magnesium to lithium (Mg/Li) concentration. A low Mg/Li shows an easier, and thus more economical lithium extraction. It is worth noting that when the Mg/Li ratio is below 6, lithium can be effectively separated by a relatively simple and inexpensive chemical precipitation method^97^. Lithium recovery can be achieved by using tri-sodium phosphate (TSP) in chemical precipitation method. For instance, chemical precipitation with TSP at 40 °C on brines with lithium concentration of 18 ppm has shown 51.62% recovery^98,99^. Mount Meager fluids show a low Mg/Li ratio in most samples, which shows its potential efficiency for lithium extraction (Fig. 9).

(d) Potassium: Solution mining is one of the applicable ways to mine potassium, and analysis of water samples from Mount Meager indicates potassium concentrations as high as 400 ppm. The deterministic model and P50 probabilistic model for reserve estimation at Mount Meager indicated production capacities of 4,187 tonnes/year and 1,081 tonnes/year, respectively.
(e) Iron and manganese: Manganese reserves are particularly low, with a P50 of just 3 tonnes/year at Mount Meager, and Canada did not produce any manganese in 2020. Similarly, the estimated iron reserves, with a P50 of 549 tonnes/year, are low compared to the 37 million tonnes/year produced in 2020 in Canada^100^.

Table 2 summarizes the results of the Monte Carlo simulation for the different sampled regions, which include thermal springs and boreholes (locations shown in Fig. 2). The values show the 50th percentile as the uncertainty bound. Boreholes exhibit high concentrations of most elements, indicating the interaction of deep, high-temperature fluid with host rocks such as quartz diorite, rhyodacite, and breccia. Elevated concentrations (mainly SiO₂ and K) in samples from boreholes near the NGF suggest that fault-associated fracture systems act as pathways for the upward migration of mineral-rich fluids. In contrast, thermal springs show lower concentrations and reserve of elements, suggesting low-temperature discharge zones of ascending fluids. Previous studies of oxygen and hydrogen isotopes at Mount Meager further support this interpretation, suggesting that shallow ground-water is primarily recharged locally due to active circulation driven by steep gradients in high-relief terrains^55^.

**Table 2:** Mineral reserve by type for each region of data collection.

	Li	Mg	Mn	SiO_2_	Fe	K
Boreholes around NGF	38.24	238.41	2.31	1200.54	363.14	1100.34
Other boreholes	46.38	881.9	2.95	485.59	19.16	313.18
Springs	4.6	113.9	2.34	475.5	1.49	216.9

Considering that the mineral transfers from rock grains to fluids during the production stage and the fracture porosity were not considered in these models, the calculated values show promising mineral reserves. Mount Meager with fracture porosity up to 40% can even contain three times more minerals within its fluid than the rock matrix porosity of up to 8%. This provides an opportunity to benefit from having a second product in addition to the energy potential from geothermal resources in the GVB.

In addition to the concentration of minerals in fluids and the potential reserve size, the economic viability of mineral recovery from geothermal fluids is controlled by (1) extraction technology and (2) commercial demand and the market price of that mineral, which are discussed in the following paragraphs.

1. *Technology*: There has been significant improvement in extraction techniques such as precipitation, ion exchange, solvent extraction, membrane filtering, and electrochemical. Each method has its own advantages and disadvantages, but they all can concentrate brine effectively and sustainably while extracting minerals. Initial attempts for mineral recovery proved a challenge in the past, but successful technologies for mineral extraction lithium and silica are in use today and continue to be developed^19,21,101^. Lithium as an example, if concentrations are as high as 50 ppm, recent technologies have significantly reduced production costs to USD 3–5 per kg recovery (compared to around USD 80 per kg in conventional seawater recovery)^102^. The operational costs can be reduced to as low as USD 1.3 when using membrane methods^16^. A summary of separation techniques to extract minerals from geothermal brine is provided in Fig. 10.
  **Fig. 10:**
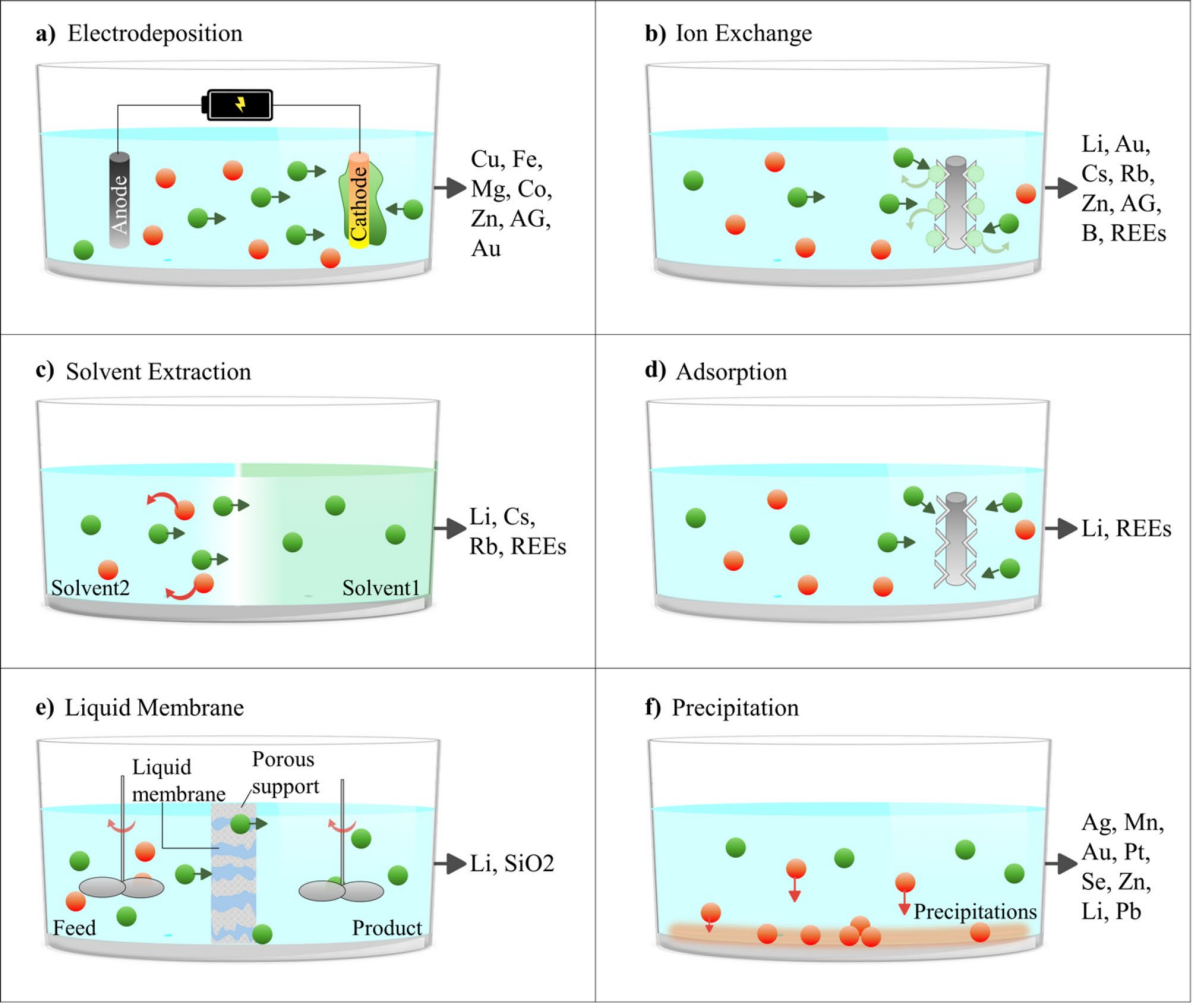
Separation techniques for mineral recovery from fluids with the list of minerals that can be extracted with each method. (**a**) An applied voltage will cause a metal to be electrochemically reduced and deposited onto a cathode; (**b**) Exchange of similar charged ions between the resin and solution; (**c**) The mineral is extracted using another immiscible solvent through a chemical process; (**d**) The adsorbent targets specific charged ions; (**e**) Selectively permit certain components across a semi-permeable membrane; and (**f**) The mineral is precipitated by adding a chemical to the fluid.
2. *Demand and price*: Environmentally sustainable extraction methods to harness minerals from geothermal brines can help Canada and the globe to meet their demand. In 2022, Canada imported about USD 473 million, USD 735 million, and USD 201 million of lithium, magnesium, and potash, respectively, which highlights the importance of using secondary mineral extraction techniques in Canada to support domestic manufacturing^2^. Several companies in Canada have started developing solution mining projects. For example, a project initiated in northeast BC to test brines from natural gas wells, suggesting the potential for economic lithium extraction alongside natural gas development^103^. Additionally, the western Canada sedimentary basin (WCSB) contains high amounts of critical minerals such as potassium and magnesium in the fluids^104^. Geochemical analyses show concentrations up to 6,000 ppm for potassium and 17,000 ppm for magnesium in the WCSB geothermal fields.

## Conclusions

As different countries decide to move to low-carbon economies and attempt to achieve net-zero targets, specific sectors can take on a critical role in the pathway to decarbonization. Canada’s transportation and electricity generation sectors, as the second and sixth largest sources of greenhouse gas emissions, respectively, play a vital role in these strategies. The future of rechargeable batteries and their respective use within electric vehicles and energy storage devices supporting renewable power generation requires critical elements. Geothermal energy is not only a green source of energy in the decarbonization pathway, but also it has an advantage as a source of critical mineral production. Geothermal energy has an opportunity for additional revenues in energy production fields that can lead to the development of geothermal resources that are not sustainable as a power generation-only field.

This paper highlights the utility of Monte Carlo simulations and appropriate probability distributions in handling uncertainties in volumetric resource calculations. This method involves running a large number of simulations (10,000 random realizations in our paper) to model the variability and uncertainties in the input parameters. The results provide a comprehensive view of the variability in the estimated mineral list. Fluid chemistry data from Mount Meager were used to assess the range of potential reserves for different minerals. It is important to note that the estimated reserve size does not include the potential contribution of minerals that exist in the rock matrix and fractured media.

In addition to producing critical minerals from Mount Meager fluid to meet Canada’s demand, extracting silica can help prevent the scaling issues that could have made a problem for Mount Meager fluid flow. Looking ahead, further considerations can include technical feasibility in the area, financial analysis, and environmental and social evaluations.

## Data Availability

The datasets used during the current study are available from the corresponding author on request.
